# Open and Cost-Effective Digital Ecosystem for Lake Water Quality Monitoring

**DOI:** 10.3390/s22176684

**Published:** 2022-09-04

**Authors:** Daniele Strigaro, Massimiliano Cannata, Fabio Lepori, Camilla Capelli, Andrea Lami, Dario Manca, Silvio Seno

**Affiliations:** 1 Department of Earth and Environmental Sciences (DSTA), University of Pavia, Via Ferrata 9, 27100 Pavia, Italy; 2 Institute of Earth Sciences, Department of Environment, Construction and Design, University of Applied Sciences of Southern Switzerland (SUPSI), Campus Mendrisio, Via Francesco Catenazzi 23, 6850 Mendrisio, Switzerland; 3 National Research Council of Italy, Water Research Institute (CNR-IRSA), Largo Tonolli 50, 28922 Verbania, Italy

**Keywords:** water monitoring, buoy, nb-iot, iot, open source, istSOS

## Abstract

In some sectors of the water resources management, the digital revolution process is slowed by some blocking factors such as costs, lack of digital expertise, resistance to change, etc. In addition, in the era of Big Data, many are the sources of information available in this field, but they are often not fully integrated. The adoption of different proprietary solutions to sense, collect and manage data is one of the main problems that hampers the availability of a fully integrated system. In this context, the aim of the project is to verify if a fully open, cost-effective and replicable digital ecosystem for lake monitoring can fill this gap and help the digitalization process using cloud based technology and an Automatic High-Frequency Monitoring System (AHFM) built using open hardware and software components. Once developed, the system is tested and validated in a real case scenario by integrating the historical databases and by checking the performance of the AHFM system. The solution applied the edge computing paradigm in order to move some computational work from server to the edge and fully exploiting the potential offered by low power consuming devices.

## 1. Introduction

Water has always been a crucial but finite resource for life with strong connections to both food and energy sectors. According to the report of the Joint Research Center (JRC) of the European Commission (EC), in order to overcome the common view of resources as individual assets, the Water Energy Food and Ecosystem Nexus (WEFE Nexus) approach has been gaining growing interest. WEFE envisages several interactions between sectors, which were largely underappreciated [1]. Water bodies such as lakes are a perfect and concrete example of what the WEFE nexus approach aims at increasing the understanding on. In fact, lakes play a central role for stocking water and make it available for different usages, e.g., agriculture, industry, drinking water, etc. Therefore, a management and protection plan to preserve ecosystems that usually exist around these key resources is also strongly needed. Nevertheless, many touristic and economic activities are bounded to lakes, making preservation also a matter of economic interests, with different stakeholders involved. Unfortunately, climate change and scarcity of water are affecting several places in the world. At the time of writing this paper, the North of Italy is facing one of the most severe drought of the last seventy years [2]. In particular, the Po watershed is dramatically under the mean level of accumulated precipitations. Such situation brings even more attention to the urgent need of finding solutions for a better water management and explore new frontiers to face the future challenges to which lakes and water resources are exposed. Furthermore, also the United Nations (UN), through their Sustainable Development Goals (SDGs), have set a dedicated goal to water quality: within Goal 6 “Ensure access to water and sanitation for all”, the indicator 6.3.2 aims at monitoring the improvement of ambient water quality since water is essential for a large number of services and needs [3]. Although the depicted situation shows how lakes are strongly stressed by climate change effects and by human activities, over the last fifty years, developed countries have been implementing a number of actions in order to increase the quality of water. Focusing the attention on Lake Lugano as the area of study selected for this research, the mitigation actions imposed by administrative bodies since the 1970–1980s worked as counteraction of the galloping eutrophication of those years [4], a process which caused plants and algae to grow in excess due to the increased availability of nutrients [5]. According to the Organization for Economic Co-operation and Development (OECD) categorization [6], lakes’ trophic status is usually classified in three ranges (oligotrophic, mesotrophic or eutrophic) with an increasing supply of nutrients and organic matter. Considering such classification, the reports of the CIPAIS commission (International Commission for the Protection of Italian-Swiss Water) highlight that, while over the last 25/30 years Lake Lugano recovered from an eutrophic to a mesotrophic status, it now appears at a standstill possibly due to unknown dynamics triggered by the changing environmental context [7]. Such scenario suggests the need for further actions in order to better understand the ecosystem dynamics and future trends and consequently react with calibrated new mitigation actions. There are two main lecture keys on which we would like to focus: the first concerns the lake monitoring while the second is related to the management of time series data. During the last few decades, environmental monitoring has been facing a deep revolution as a consequence of new technologies spreading across the market (e.g., the Internet of Things—IoT; low cost sensors; and new data transmission methods [8,9]). Such revolution is part of a wider process called digitalization [10] that is impacting all economic sectors as well as human life. The new technologies, mentioned above, could potentially contribute to the densification of official monitoring systems, generally maintained by public administrations, which strongly need to improve the monitoring network for various scopes and with a different level of accuracy to support local needs. By way of example, the Swiss hydro-meteorological monitoring network includes a first level network, whose activity is focused on monitoring climatic variables with highly accurate sensors and tools and a second level network composed by small regional monitoring systems (one per canton), with more dense stations to guarantee effectiveness in supporting the local water management and in handling hazardous phenomena [11]. On the basis of such new innovations, it is common to find in literature several papers trying to solve research problems by building custom devices for monitoring environmental parameters [12,13,14]. These devices are mainly based on micro-controllers such as Arduino, Raspberry or Pycom solutions and make use of low cost sensors and modules to keep the costs of the system competitive [15,16]. If, on one hand, such an approach demonstrates high potential and good results [17,18], on the other hand, it could often lack in proved data quality replicability and in clear documentation to guide the user in building the system (“how to”). Furthermore, while high technical skills are required, in most cases, these solutions do not permit the deployment of a complete infrastructure chain that starts from data collection and ends with data management, comprising tools for data analysis, data quality control, notifications, etc. The availability of a tested and replicable open digital ecosystem composed of all these elements is crucial to increase the adoption of such solutions for the implementation of reliable real working monitoring systems. One of the reasons for the described situation could be related to the budget of research, which is usually limited considering their need to solve specific issues. In addition, focusing on water lake monitoring, periodic campaigns (i.e., weekly or monthly) are usually performed using on-board instruments (e.g., CTD probe measuring conductivity and water temperature or Secchi disk to measure transparency) and limnological vessels which are then further analysed in the laboratory [19], while a real-time automatic monitoring network is generally missing. In literature, there are examples of advanced systems that can improve the information gathered using traditional methods, but they are usually very expensive and strongly oriented to scientific experimental research [20,21]. On Lake Geneva, a floating laboratory is installed, hosting more than 30 scientific projects with the aim of measuring and modelling key physical and biogeochemical processes at high frequency [22]. Data management and integration is the second key issue that this paper would like to focus on. Unfortunately, such processes are facing a slow adaptation by the public and private sectors [23] in some of the water fields. For example, in the context of data management, a report on digital transformation in the U.S. water utilities sector by Dodge Data and Analytics highlighted that 90% of respondents to the survey confirmed that their data lie in disconnected systems such as spreadsheets, paper records, etc. Moreover, even digitized data often lack in uniformity of formats, interoperability and integrity. This situation is even more problematic in developing countries where the digitization and consequently the digitalization processes are still far from being in an advanced status [24,25].

The possible reasons hampering the adoption of new technologies in lake monitoring and digitalization of the water sectors can be synthesized with the following points [26]:

- lack of digital expertise: the demand of hybrid professional profiles is growing in the market, especially in the environmental sector and more generally in data science [27,28];
- resistance to change: human resources are often reluctant to change their mindset and way of working [29];
- lack of data interoperability between systems: this is a particularly delicate topic, as in the environmental system there are usually many proprietary solutions, each with its own standards for data collection, logging, transmission and sharing. This very wide topic concerns architecture, protocols and standards [30];
- lack of an easy-to-play solution: this aspect is strongly connected to the previous one, since the problem of having different kinds of proprietary solutions highlights the lack of a simple and smooth way of importing and integrating different data formats in a single solution;
- costs: it is a common belief that the benefit or value of having data in real time and with high frequency cannot outweigh the initial investment and maintenance costs [21].

In this context, this research would like to explore if an integrated and fully open software and hardware solution could address the limitations mentioned above and foster digitalization in the water sector. In fact, if successfully adopted, such a solution could help in understanding how water ecosystem management could benefit from the advantages of digitalization and in tackling the local effects of climate changes thanks to the support by high-frequency monitoring systems in discovering yet unknown dynamics. In particular, for testing and validating the system, we focused on the Primary Production (PP) estimation of the lake Lugano (the area of the study), a parameter which is generally calculated for the surveillance of the lake eutrophication process and that represents one of the most important indicators evaluating the lake metabolism. The PP can be defined as the process of assimilation of inorganic carbon into organic matter by autotroph organisms. In order to monitor the situation of Lake Lugano, twenty water samples are collected using little glass bottles at different depths, each at a distance of about 1 m from the previous one (from 0.5 to 20 m depth). Such samples are then analysed following the Nielsen and Schindler method [31,32], whose results are processed using an algorithm that calculates the yearly amount of Carbon for squared meter (gC/m^2^). Those values are finally used by administrative bodies to evaluate the status of the reservoir. Unfortunately, this methodology presents some critical aspects that can be synthesised with the following points:

- it is an expensive procedure since it requires costly external analyses and manpower/working hours;
- highly qualified personnel is involved during the campaign, with very high cost per hour;
- results are based on a low temporal resolution time series (monthly samples);
- during the sampling process, a potentially dangerous radioactive component is manipulated by technicians.

For these reasons, an Automatic High Frequency Monitoring (AHFM) system was developed in order to apply, test and validate new technologies and recent methodology such as the PP estimation based on a number of parameters that can be measured in real time and high frequency using sensors (e.g., dissolved water oxygen, solar radiation, wind speed, water temperature) according to the Staher et al. method [33]. This approach calculates lake metabolism rates on a daily time scale. Since it can potentially replace the traditional method but it is still unfortunately still underused, it must be highlighted that, in order to consider this new approach as a valid alternative, a wider in-depth investigation should be conducted in the future, in addition to the analysis of technological aspects on which this article is focused.

## 2. Materials and Methods

The existing limitations that block the digitalization process may be overcome by the development of a fully monitoring system (from data collection to data management), which follows some requirements, such as:

- use as much as possible an open source approach either for the software or for the hardware part;
- use open standards for data integration and sharability;
- maximise the possibility of easily extending and customising the software;
- use cloud technologies to deploy server side components.

Once the different parts of the system were developed, they were tested and validated in a real case scenario. In order to make things initially simpler, we focused on a specific problem that affects the selected study area and then verify if the proposed solution could effectively improve the ongoing situation. Since an all-in-one commercial solution does not exist according to the authors’ knowledge, a fully open and replicable solution is proposed in this article.

Such activities were conducted in the framework of the SIMILE (Informative System for the Integrated Monitoring of Insubric Lakes and their Ecosystems) INTERREG Italy-Switzerland project, which aims at integrating different techniques (in-situ monitoring, remote sensing and citizen science) to monitor trans-boundary and sub-alpines lakes such as Maggiore, Como and Lugano.

### 2.1. Area of Study

**Figure 1:**
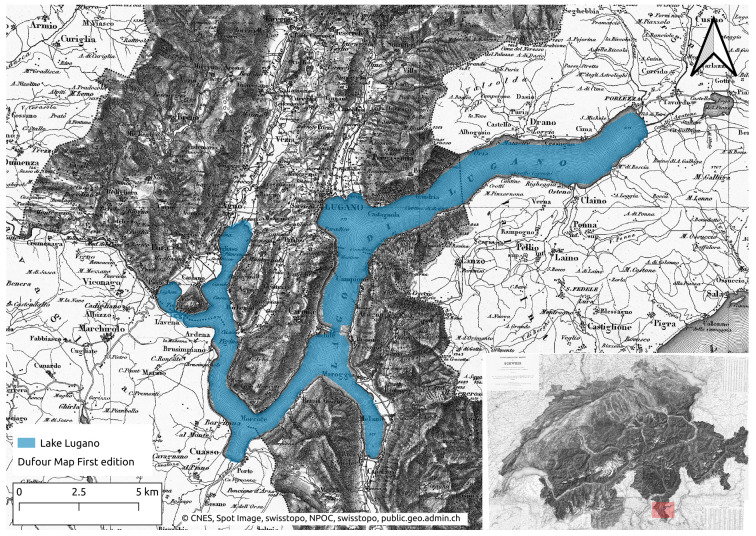
Map of Lake Lugano, southern part of Switzerland.

The study area individuated to test and validate the monitoring system is Lake Lugano. It is composed of three sub-basins (see Figure 1). The North and South basins are divided by the Melide bridge, which was built on a sublacual moraine. The third small basin is located in proximity to the Tresa river. From a morphometric point of view, the Lugano South basin has an area of 21.4 km^2^ and a mean and maximum depth, respectively, of 85 m and 89 m (Table 1). This study is focused on the South basin because of the main interest due to its precarious water oxygenation, which, during the winter season, can reach the anoxide status in the deepest part of the lake. This is one of the negative effects of the euthrophication process started during the 50 s with a peak in the 80 s. Thanks to the European Directive (2000/60/CE and 2006/7/CE) and to the Switzerland law (Federal Act on the Protection of Waters and Water Protection Ordinance), the situation is improving, but it is still far from the fixed goal of 150 gC/m^2^y (mean average of 300 gC/m^2^y of last 5 years) [4,34]. Hence, Lake Lugano has been constantly monitored since the 1970s through manual campaigns which occur one or two times per month depending on the type of monitoring (chemical, physical or biological).

### 2.2. The System Architecture

**Figure 2:**
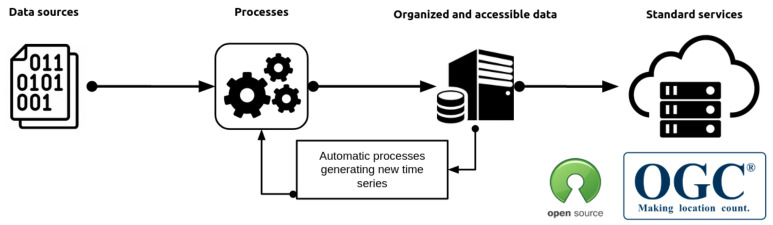
The data flow architecture.

The AHFM system is part of a wider architecture that covers all the components engaged in the monitoring system. To this end, the first step concerned the research and collection of all the historical time series of Lake Lugano which are usually archived in different data formats (e.g., CSV or TXT file, Microsoft Access databases or Microsoft Excel files, etc.). One of the main objective was to integrate data sources into a single sink to obtain a unique entry point and simplify a situation dominated by a lack of integrity, interoperability, uniformity as well as being prone to the errors process because many pieces of data are replicated in different data formats. In Figure 2, the schema of the data flow is presented; different data sources were integrated into an organized and accessible system that can make them available to stakeholders using standard services. In addition to the reorganization of the existing datasets, which is continuously updated by the data collected during the monitoring campaigns, new observations were generated in quasi real-time by the AHFM system. Besides the development of components that can integrate and import already existing data, the system relies on the Sensor Observation Service (SOS) of the Open Geospatial Consortium (OGC) for data sharing and interoperability. It is a web service that allows for query environmental data encoded using the Observations and Measurements (O & M) model coming from sensors or more generally by a so-called “procedure”. The metadata of each *procedure* are formatted following the Sensor Model Language (SensorML). In addition to the SOS OGC standard, data and metadata are available through a REST API interface in order to better link web applications. In summary, the approach proposed permits retrieving observations using a single point access and further elaborate on them to create new time series.

During the development of the proposed architecture, of which components are described in detail in Section 2.4, three main approaches are used: the Service-Oriented Architecture (SOA); Edge Computing (EC) and WebAssembly. The first is a development pattern where a service is a component application that communicates to the other through a communication protocol over a network [35,36]. In SOA, services play a key role and become the basic computing unit to support development and composition of larger, more complex services, which in turn can be used to create flexible, ad-hoc and dynamic applications [37]. The second (EC) is a computing paradigm that brings data elaboration and storage closer to data sources such as in some IoT devices [38,39]. Finally, WebAssembly provides a way to run low level code written in multiple languages (Rust, C++, C#, etc.) on the web at near native speed using a modern web browser.

### 2.3. The Automatic High Frequency Monitoring (AHFM)

**Figure 3:**
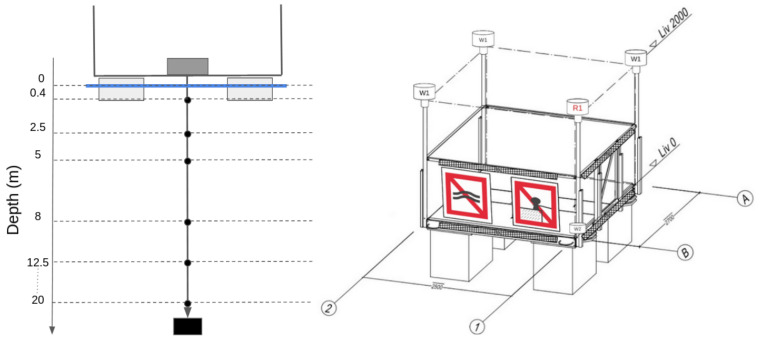
On the left, the design of the position of the chain sensors; on the right, the project of the platform designed and realized by Officine Ghidoni SA (Via al Pizzante 9, 6595 Riazzino, Switzerland).

The already cited AHFM is developed and then mounted on a platform of 2.5 × 2.7 m in the middle of Lake Lugano Southern Basin (45.95866° N, 8.89371° E, EPSG 4326). In Figure 3, the design of the platform is presented. It is composed by a metallic structure sustained by four floats and anchored to the bottom of the lake with four chains connected to concrete blocks. At the middle of the floor, there is a hole where a chain of sensors can be lowered. The purpose of this structure is to offer space and support to easily install and maintain the hardware components used to monitor the water environmental parameters.

**Figure 4:**
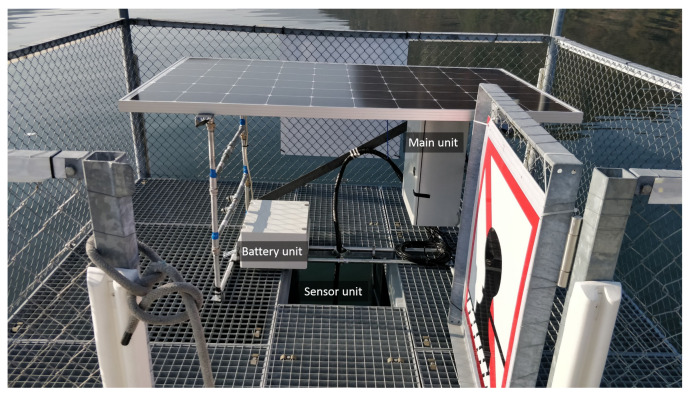
The AHFM system architecture.

The AHFM system, mounted on a customized structure fixed on the center of the platform, is composed of three main units (Figure 4): the power unit; the sensor unit; and the main unit.

#### 2.3.1. The Power Unit

**Figure 5:**
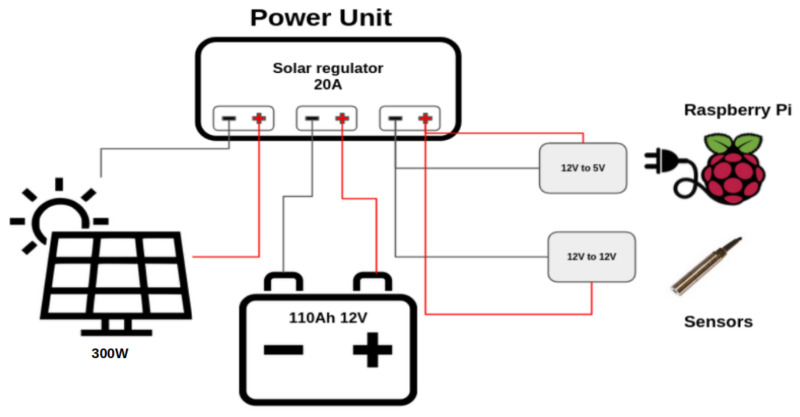
The power unit.

The power unit consists of a 12 V per 110 Ah battery (1320 Wh), a 20 A solar regulator and a photovoltaic panel with an estimated peak power of around 300 W. The solar regulator provides 12 V to the system voltage regulator, which then steps down the current to 5 V to power up the core unit, and to 12 V to power the sensor unit (Figure 5).

#### 2.3.2. The Sensor Unit

**Figure 6:**
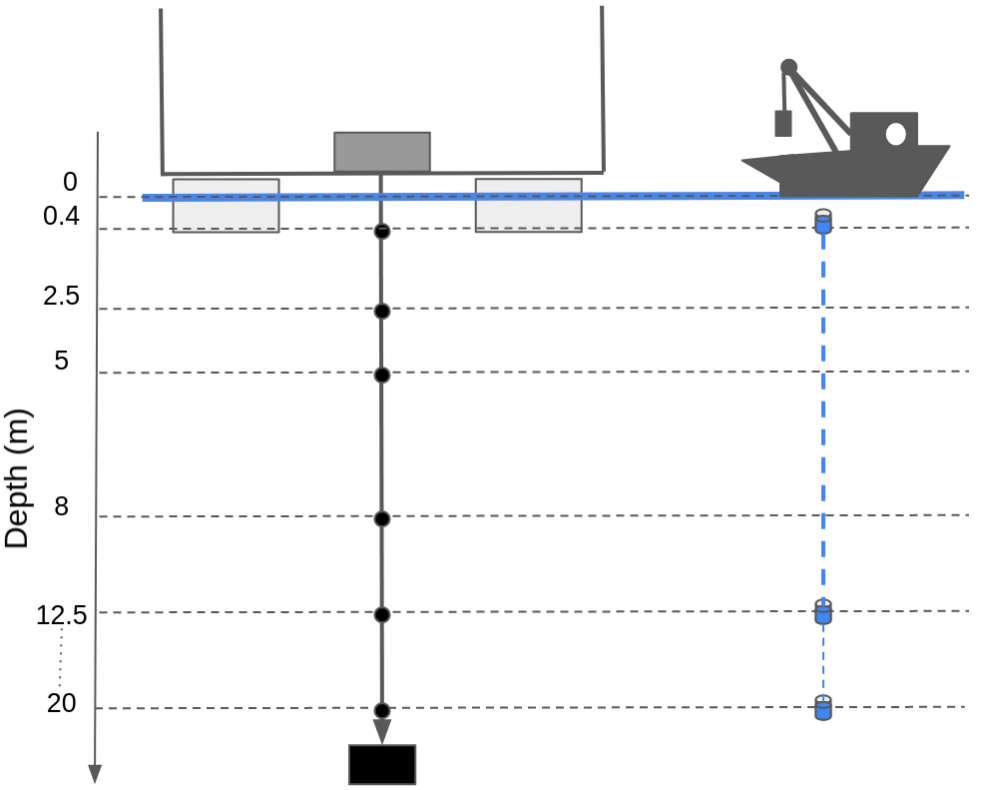
Comparison between AHFM and manual sampling depths.

The sensor unit relies on six Optical Sensors based on luminescent technology (OPTOD) by Ponsel, which measures the dissolved oxygen in terms of concentration (ppm and mg/L) and saturation (%) with an accuracy of, respectively, +/−0.1 ppm or mg/L and +/−1%. In addition, it provides the water temperature, which is used to estimate the saturation. The OPTOD sensor can be connected to any type of datalogger through the Modbus RS-485 connection since it has an open protocol for command and data transmission, and it requires 5–12 V current. The sensor is factory calibrated. The re-calibration process can also be performed, putting the sensor at open air checking that it measures 100% of oxygen saturation. Otherwise, when this process does not correct the measurements, a proper calibration should be performed in the laboratory or delivering the sensor to the main factory. The six sensors were assembled in order to form a chain in which each sensor is positioned at different depths (0.4, 2.5, 5.0, 8.0, 12.5 and 20.0 m). The depths correspond to the position where the traditional water samples are collected during the monitoring campaign performed by limnologists (Figure 6). They usually take one sample every 0.5 m, so the most significant point is chosen with a higher density in the first five meters since the productivity is concentrated in the photic area [40].

#### 2.3.3. The Main Unit

**Figure 7:**
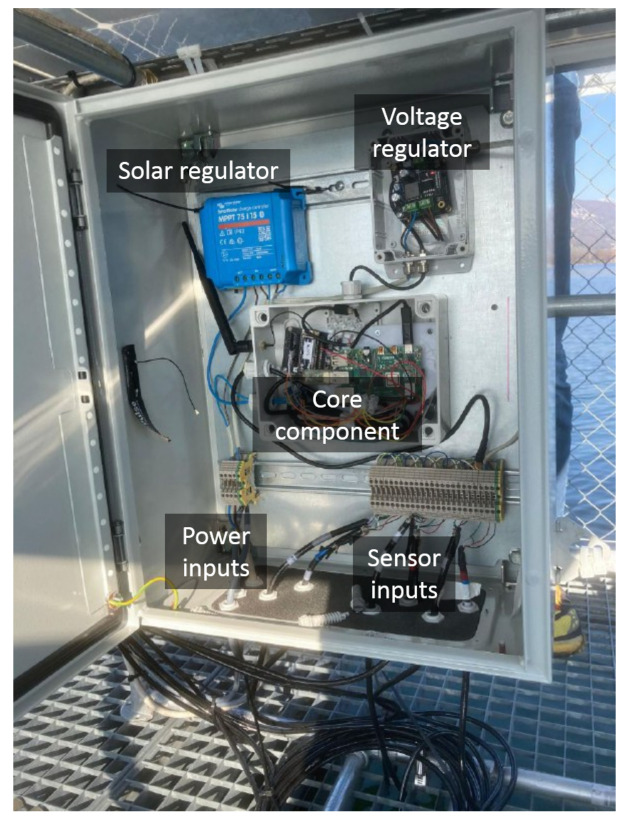
The main unit components.

The main unit (Figure 7) includes solar and voltage regulators, a section with terminals for sensors and power inputs and finally a core box. The power inputs concern the connection with the power unit based on a miniature computing platform based on the Raspberry Pi 4 model. Raspberry 4 is chosen instead of other MicroController Units (MCU), like Arduino or PyCom devices because of its larger computational power, its flexibility and acceptable power consumption which therefore permit to perform some in-built analysis directly at the edge of data source as for the EC computational paradigm. The workflow implemented in the control unit is developed using the Python programming language, and it is available at the github repository under the GNU GPL v3 License (https://gitlab.com/danistrigaro/station_configurator/, accessed on 1 August 2022). The algorithm obtains data from the sensor unit every minute through a MODBUS RS-485 interface, performs some quality checks on the data and aggregates them every 10 min. After the aggregation, the data are sent to the server infrastructure. The control unit supports the SOS standard for data sharing thanks to the istSOS software, a lightweight data management system which is successfully used for various applications based on the hydro-meteo weather stations data of the Canton Ticino [41].

The communication unit is based on a IoT shield which sends data through NB-IoT or LTE-CATM1. The data transmission takes advantage of the MQTT protocol which is very light and widely used in IoT systems. The NB-IoT or LTE-CATM1 are selected instead of the LoRa communication because they have more bandwidth for data transmission while maintaining very good performance in power consumption.

#### 2.3.4. The Firmware Structure

**Figure 8:**
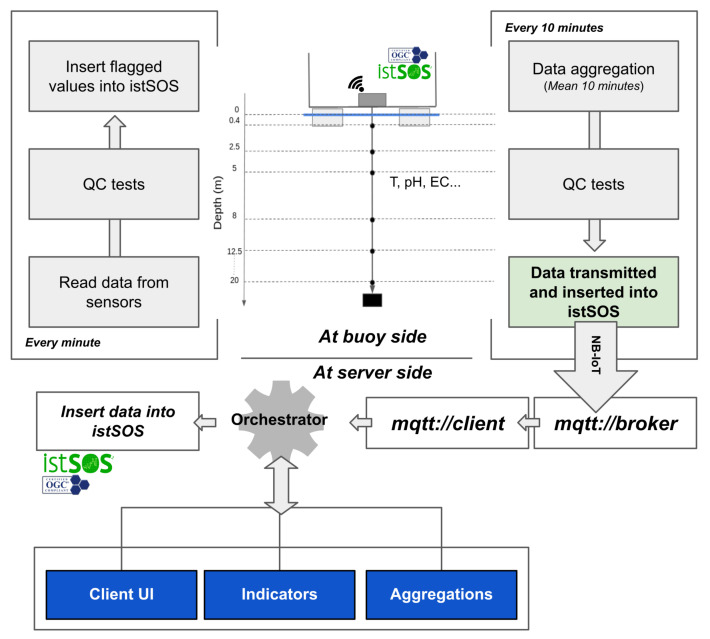
Data flow from buoy to server.

The control unit, based on the Raspberry pi 4, hosts a Raspian Pi OS Lite distribution, which was customized in order to minimize the current consumption disabling the unused features. Therefore, istSOS, an implementation of the SOS standard, was installed to manage on the edge all the data collected using the SOS standard. The availability of a powerful device that manages data can help in performing some tasks that otherwise are usually performed on server side. In fact, the data flow (see Figure 8) concerns the collection of raw data from the chain of sensor every minute; then, these data are instantly checked to avoid null value and outliers by controlling that the value is in the range specified in the sensor datasheet. Data are inserted in the local istSOS which will be assigned a flag to indicate the quality of the value. Every 10 min, a script is run to obtain the last 10 min data, to check the consistency of data in terms of variability between them, and finally to aggregate only the data which passed the tests. If the data flagged as correct are less than 60%, then the aggregate value is flagged with a quality index of 200, otherwise of 201. According to the configured sending time, the aggregated data are transmitted to the server side using NB-IoT through the MQTT protocol. If data are correctly sent to the broker, the flag of each observation is updated on local istSOS in order to be able to filter between sent and not sent data.

The data flow was implemented using custom Python scripts that are automatically generated running the initial configurator script that reads the YAML settings file and performs the following steps:

- check the availability of the configured sensors;
- register sensors in local and remote istSOS;
- create the read and write script that collects raw data from sensors based on the frequency set in the configuration file;
- create the aggregation script that collects raw data from istSOS and aggregate them using the frequency set in the configuration file;
- generate the Python script which obtains the aggregated data from local istSOS, activates the NB-IoT module and transmits each single observation to the remote istSOS.

The execution of the scripts is possible because, during the configuration process, scripts are added to the crontab schedule according to the specified frequency.

Once data are transmitted to the server, they are available for further validation processes, analysis, reports creation, etc.

### 2.4. The Data Management

**Figure 9:**
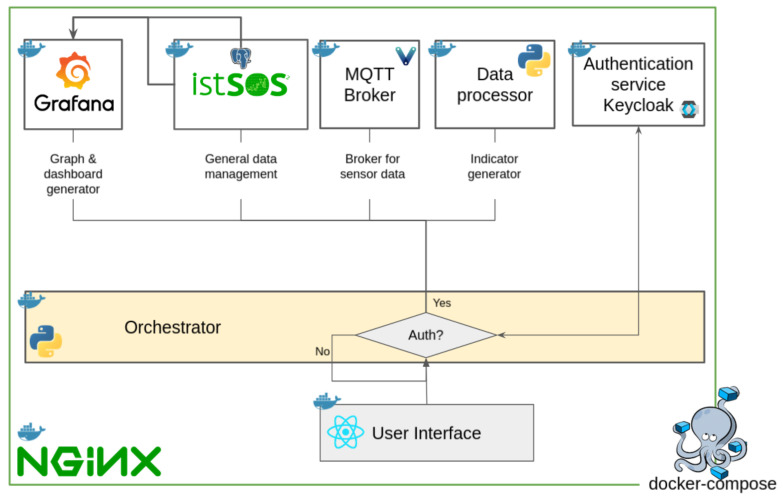
The system architecture.

The software architecture (Figure 9) is designed to store, share and elaborate the data coming from the AHFM system and from the historical time series collected by the traditional campaign since the 1970s. It is composed by seven services (Figure 5): a web interface; an orchestrator (Python); an authentication service (Keycloak); a database (https://www.postgresql.org/ (PostgreSQL), accessed on 1 August 2022); a data management system (http://istsos.org/ (istSOS), accessed on 1 August 2022); a graph and dashboard generator (https://grafana.com/ (Grafana), accessed on 1 August 2022); an MQTT broker for AHFM system data (www.vernemq.com (VerneMQ), accessed on 1 August 2022); a processor for indicators’ elaboration (Python); a user interface (https://nextjs.org (Next.Js), accessed on 1 August 2022). The implementation of each single service was done using Docker technology. In this way, it was possible to create a Compose file to automatically generate the services. Compose is a tool to define and run multi-container Docker applications.

#### 2.4.1. Data Sources and Standards

In order to facilitate the decision makers through a synthetic interface based on dashboards and graphs, all information needs to be digitally accessible from a unique entry point. Therefore, all the data sources are initially grouped, then processed and digitized. Basically, four main sources exist:

1. data from manual sampling campaign based on sensor probes;
2. data from laboratory analysis performed on water samples;
3. data from satellites;
4. data coming from a real-time monitoring system.

To foster the data integration and exploitation, as well as data sharing, international standards are used as a powerful tool which provides a predefined language to increase the interoperability between data. In the environmental field, the Open Geospatial Consortium (OGC), founded in 1994, is a community which collaboratively develops open interface standards and associated encoding standards, and also best practices to exchange geospatial information. To enable data integration, the Sensor Observation Service (SOS) [42] open standard is selected to handle all the data available.

#### 2.4.2. Support for Profile Data Type

**Figure 10:**
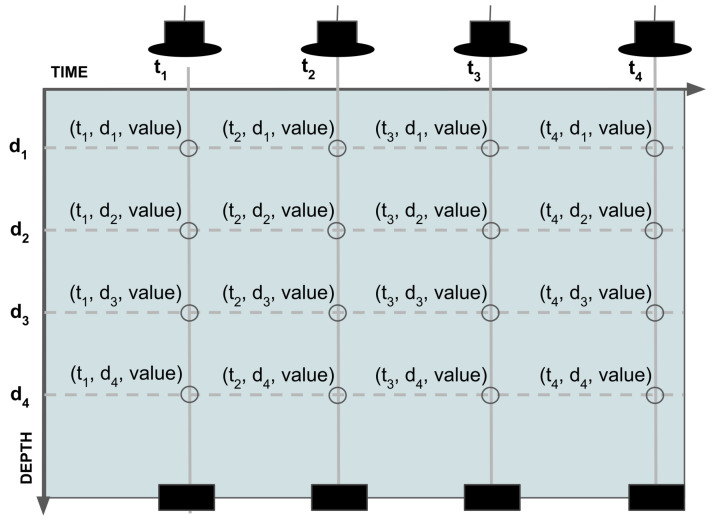
Profile data type.

The profile data are a particular time series where, for each datetime, an array of data composed by depth and value is available. Instead of a bi-dimensional data representation (usually time and value), in profile data, there are three dimensions: the time, depth and value (Figure 10). For this reason, istSOS and Grafana, two of the selected applications which shape the server architecture, do not natively support the profile data type. In fact, istSOS, which was mainly used for time series data, it originally does not support the data profile format. To fill this gap, in this work, the software has been further improved by implementing a feature to support data profile type, which is then incorporated into the core of the software. The Grafana application has the function to generate dashboards and plots. In addition to istSOS, it is an open source software which allows for query different data sources and visualizes the data requested in many ways. It is easily extensible thanks to the possibility to develop plugins in order to add new data sources and new panels for data representation. Hence, a new data source plugin has been developed in order to enable the communication to the istSOS instance and a new panel to visualize the data profiles.

### 2.5. Data Quality Control Process

The data Quality Control (QC) in a monitoring system aims at detecting erroneous or suspicious values which could be a sentinel of possible system malfunctions. Once detected, they should be automatically or manually corrected or deleted [43]. In Table 2, a list of Quality Index available on istSOS is used to flag values. Higher values correspond to a higher level of quality. The second digit of the QIs is used on the edge to understand if data are correctly transmitted to the MQTT broker (digit 1) or not (digit 0).

The QC tests implemented follow the WMO guidelines [44]. Tests are applied in real-time at the edge and in post-processing at the server side.

#### QC Process at the Edge

The AHFM system collects new measures that undergoes two tests before being stored in istSOS. The first test consists of verifying that the measure is a valid number (is not a “Not a Number”, NaN). The second applies a range test, to identify gross error and outliers (Table 3). The range test is based upon specifications and limits found in the sensor’s datasheet.

Once it is time to aggregate data, two other tests are performed. The step test (Equation (1)) controls the variation compared with the previous and the next observed values not being above a prefixed limit [44]:

(1) $$|V_{i}-V_{i-1}\left|+\right|V_{i}-V_{i+1}|\leq \sigma_{v}$$

The second check performed (persistence test) controls the fact that between values there is a minimum variability. This test is performed to detect possible sensor malfunctioning that often results in a returned constant value, which is not corresponding to the current observed phenomena. While other methods are proposed in literature by [45,46,47], we implemented those proposed by [48] (Equation (2)):

(2) $$AVG=\frac{\sum_{i=1}^{n}V_{i}}{n}V_{i}\neq AVG$$

The average is then performed only on the values that passed such tests. If less than 60% of the values retrieved for the aggregation do not pass the tests, the value aggregated will be flagged with a lower QI flag.

## 3. Results

Hereinafter, the presented results refer to an entire year of data collected from 1 January to 31 December. The system was implemented and started the data collection in November 2020, but we considered the first two months as a testing and stabilization phase where small improvements and fixes were applied.

### 3.1. Data Completeness

**Figure 11:**
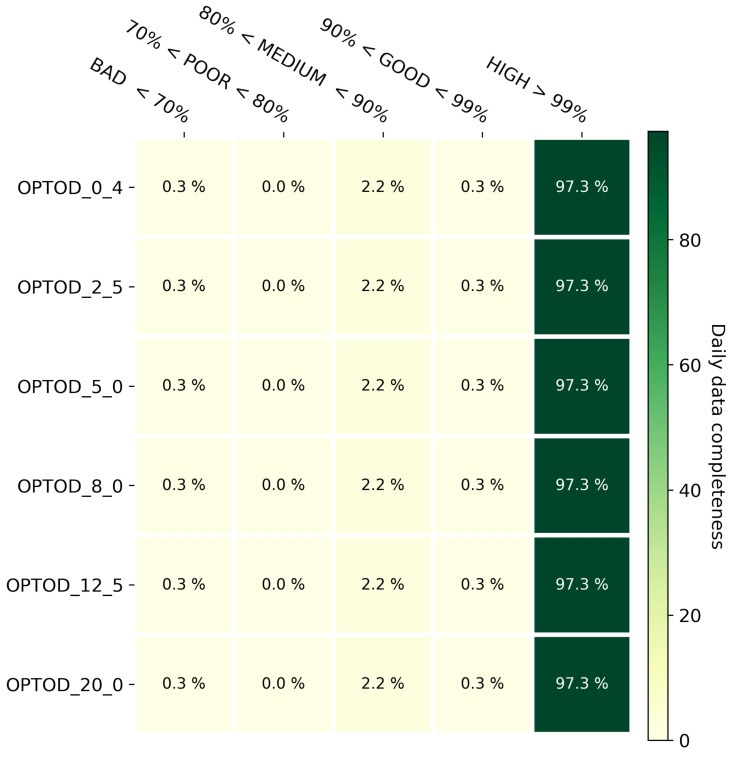
Daily data completeness heatmap based on the classification of the daily percent value of completeness in five FL classes: bad, poor, good, and high.

The evaluation of data completeness is an important estimation of the reliability of a newly developed system. Basically, taking into account the whole period of work (from the 1 January to 31 December 2021), the amount of the data expected, calculated from the sampling frequency, is compared to the amount of data correctly archived. Since raw data are collected every minute for a total of 1440 observations per day, the daily archived observations were counted and then the daily percentage of completeness was calculated. These values are again grouped into five categories: bad if the percentage is less than 70%; poor if the percentage is less than 80% but more than 70%; medium where the value is between 80% and 90%; good between 90% and 99%; and finally high, where the percentage is more than 99%. The graph in Figure 11 shows some results for all the sensors installed even if each sensor has its own connection and data are read from them one piece after another. Only 0.3% of the daily data completeness is categorized as bad, 2.2% in the medium class and finally 97.3% of the values has a daily percentage of completeness greater than 99%.

### 3.2. Data Quality

**Figure 12:**
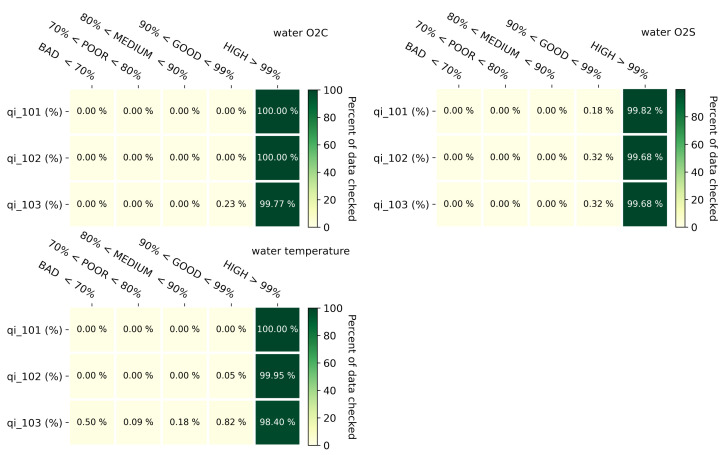
Daily aggregated data quality tests categorized into five FL discrete classes.

In this section, the results of the quality control process described in Section 2.5 are shown. In Figure 12, a heatmap per each observed property showing the result of such an analysis is proposed. In those graphs, data were aggregated per day by making a percent of data that passed a specific test. Then, each day is categorized according to five classes which can describe the percent of days that have bad, poor, medium, good, and high quality. Water oxygen concentrations expressed in ppm and mg/L have exactly the same results since they are basically the same measure but expressed with a different unit of measure. Almost 100% of the values passed the range and step test. Just 0.23% of the day were categorized with a good level of quality, which means that the remaining 99.7% have a high level of quality. A similar behavior is registered for the water oxygen saturation. In addition, 99.82% and 99.68% of the days have respectively more than 99% of data that passed the range test and the step test as well the persistence test. The last graph in Figure 12 is about the water temperature. The quality control process shows that 98.4% of day was categorized with a high level of quality since more than the 99% of the values of those days passed three tests.

### 3.3. Maintenance and Calibration

The system deployment also coincided with the start of the maintenance activity, which is very important in particular when sensors are submerged in very productive lakes such as lake Lugano. During the winter, the maintenance does not highlight specific issues. Starting from the spring and especially in summer, sensors were covered by an algae film which could affect the measures of the dissolved oxygen. If on one hand the maintenance is focused on cleaning the sensors, during July, a communication issue blocked the data transmission, but not the data collection, which was regularly archived both on istSOS and on a text file on a USB stick. The flash drive was introduced after a few months for securing eventual SD card failures in order to have a data backup available.

**Figure 13:**
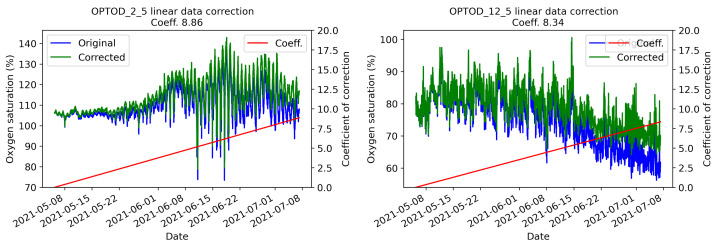
Data correction of time-series collected by the OPTOD sensors by Ponsel one positioned at 2.5 and another at 12.5 m depth.

In addition, during the maintenance activity, sensor calibration is verified by controlling the fact that measures reach a value near 100% of oxygen saturation in the open air. To this end, the chain of sensors is pulled out of the water waiting for them to be dry in order to prevent erroneous measures that could be affected by the presence of the water. If values deviate for more than ±5% from 100%, the sensor is re-calibrated applying a coefficient of correction which could be positive or negative. This process is performed by connecting the sensor with a laptop and by using the Calsens software. The coefficient is used in post-processing to correct the previous data collected by applying a linear regression between the last calibration and the new one. In Figure 13, an example of the data correction performed on sensors positioned at 2.5 and 12.5 m depths is shown by overlapping the raw data with the corrected one. In these cases, graphs highlight the fact that the sensors have generally a drift which causes an underestimation of the values. During the period considered, sensors generally suffer from an overestimation of the values. Considering that the platform was visited eight times to perform maintenance, the calibration coefficient was negative only two times, showing an overestimation of the values. From the experience gained during the working period of the AHFM system, in a highly productive lake such as Lake Lugano, which has a mesotrophic status, a maintenance program consisting of a visit every two weeks is enough to guarantee a good quality of measurements and a good status of the chain of sensors. Each visit lasted an average of three hours, but since this value comprises developing and testing actions, it is possible to consider one hour an half for a standard maintenance activity (cleaning sensors and solar panel, calibrating sensors if needed and downloading raw data).

### 3.4. The Powering System Data

**Figure 14:**
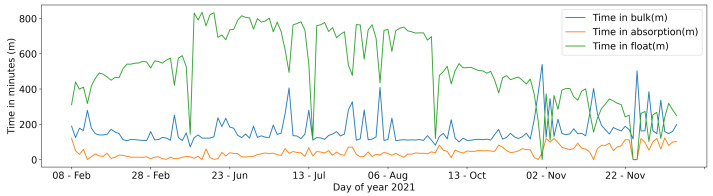
Time in bulk, absorption and in float.

Data collected to analyse the power management are retrieved from the Victron Energy solar regulator. Such data do not cover the entire year of analysis because data were not always downloaded from the device during the maintenance activity. The power system is based on three type of phases: bulk; absorption; and float. The bulk is the phase where the charger sends current and amperes to the battery so that the battery voltage rises. The bulk stops and absorption starts when the battery is full and reaches the specified absorption voltage. If the battery is full and the photovoltaic panel is in a situation to transform solar energy in current, the float phase begins with the simple job of keeping the battery full. In Figure 14, the trend of the three described phases is highlighted. The time in float has a peak during the summer of 800 min and decreases in cold seasons to a minimum of about 100. The time in absorption and in bulk are quite constant, respectively, around 160 and 40 min.

**Figure 15:**
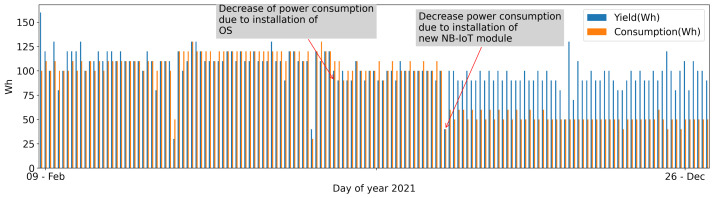
Energy yielded vs. energy consumed by the AHFM system.

In Figure 15, the comparison between the yield and the consumption energy expressed in watts per hour is shown. Even though the initial consumption is estimated to 110 Wh per day, the installation of a new Operating System, which is more optimized in power management, and the change in the communication hardware module, brings a drastic decreasing of the power consumption.

**Figure 16:**
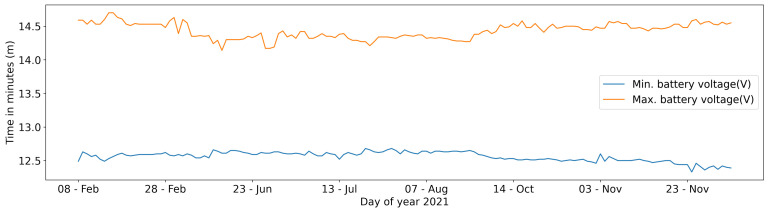
Minimum and maximum battery voltages.

A final consideration on the power management system concerns the trend of the voltage battery which rarely reaches values under 12.3 Volts (Figure 16).

### 3.5. Comparison between AHFM System and IDRONAUT’s Probes

**Figure 17:**
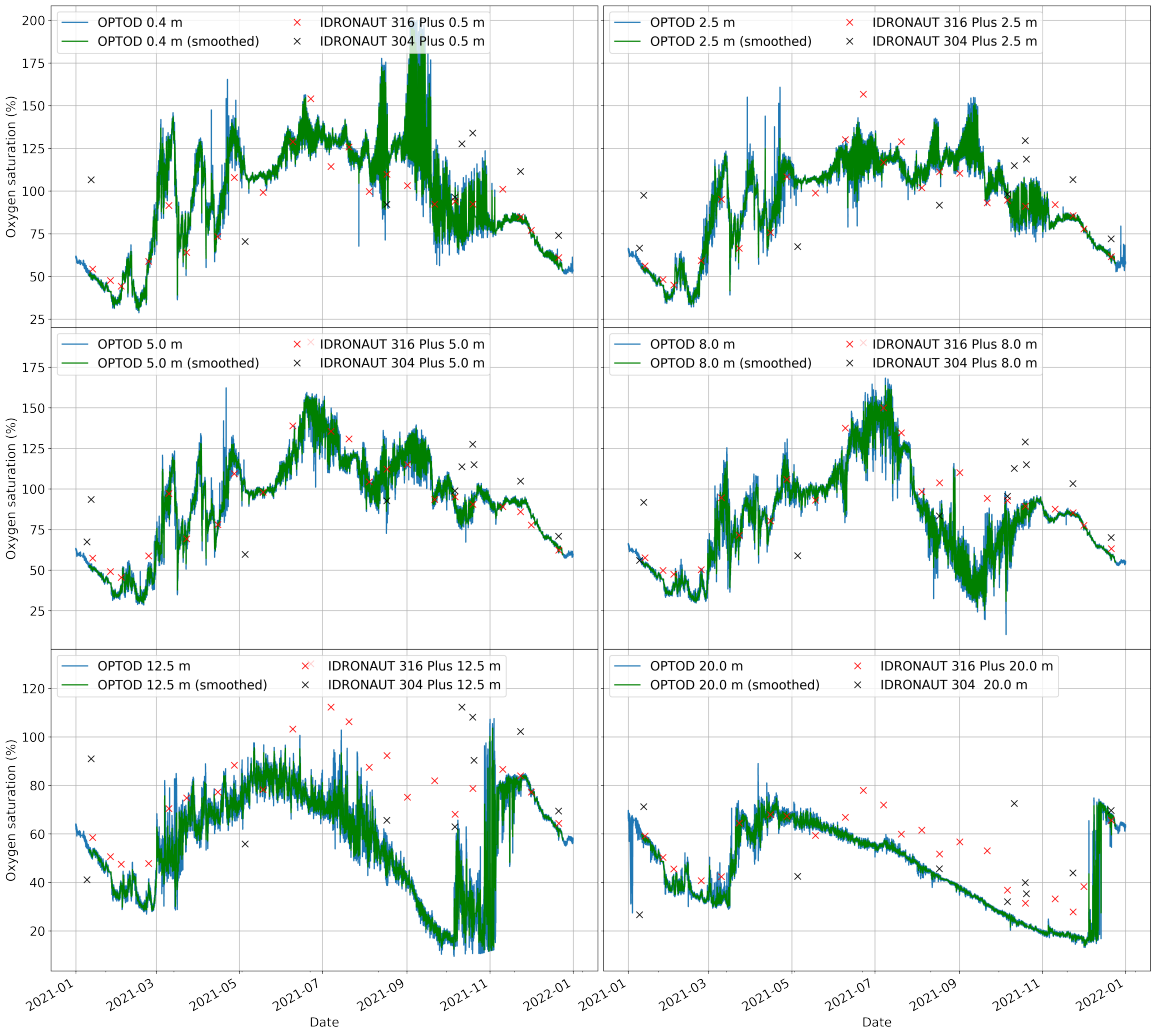
General overview of the time-series comparison between AHFM and IDRONAUT probes.

The developed system permits on one hand to collect data in real-time and on the other to import all the historical time series coming from the traditional campaigns in a unique entry point. To this end, the data management software, at the server side, helps with homogenizing data and metadata as well as in enabling the comparison and validation of the AHFM system dissolved oxygen data. In Figure 17, a general overview of the AHFM dissolved oxygen time-series is compared with the measurements taken with two types of probes. In fact, during the traditional campaigns performed in 2021, water profiles were taken using an IDRONAUT 316 Plus and an IDRONAUT 304 Plus probe. The first is a multi-parametric piece of equipment that can measure water temperature, water oxygen, Chlorophyll-a and Phycocyanin concentrations. The second is an older instrument which measures water temperature and dissolved oxygen. These instruments are used to make water profiles generally two times per month. The output is a time-series with a low temporal resolution but a high spatial resolution since data are recorded every 0.5 m by manually dropping down the probes. In Figure 17, a general overview of the comparison between IDRONAUT’s probes and the AHFM system time-series is shown. Focusing on the first four graphs, which respectively concern data collected at 0.4, 2.5, 5 and 8 m depths, a similar trend can be highlighted. From January to February, a negative gradient of the water oxygen saturation is followed by an increment which was registered by all the sensors. This event is followed by another, a more marked change, that began during the end of February. Two other peaks respectively near the end of March and the end of April follow. Such events can be well recognized at 0.4, 2.5, 5 and 8 m depth. From May until the beginning of July, a positive gradient is constantly registered from 0.4 to 8 m. It is more vigorous at 5 and 8 m depths. After that, the water oxygen saturation decreased until the second week of September. From 0.4 to 5 m depths, such trend is hindered by temporary increases in the values. In particular at 0.4 m, very high variations are measured with peaks that reached 200% of saturation. While at 0.4 and 2.5 m high variations are not associated with a general increment in the trend, at 8 m depth, a positive gradient starting from the second week of September until the beginning of November was detected. The last remaining part of the year is characterized by a negative gradient with the absence of sensible low and high peaks. At 12.5 and 20 m depth, the behaviour of the saturation of the dissolved oxygen is completely different from the previous description. At 12.5, there is a bell-shaped trend with a peak in May and a minimum in October. Afterwards, values are characterized by very high variations, but, in November, they became more stable and began to decrease at the beginning of December. At 20 m depth, the maximum is reached during April; then, a continuous decreasing of the saturation was detected till the end of November or the beginning of December, where, on the contrary, values started to increase to 70%. Values around 60% were then registered during the end of the year.

**Figure 18:**
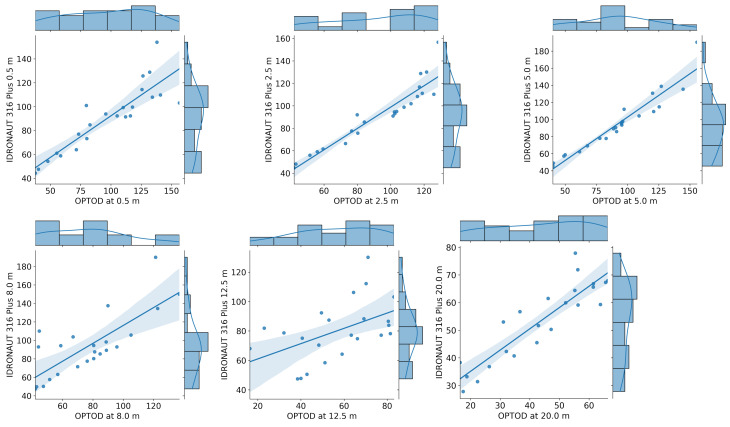
Point to point comparison of dissolved oxygen saturation time-series between AHFM and manual sampling with an IDRONAUT 316 Plus probe.

**Figure 19:**
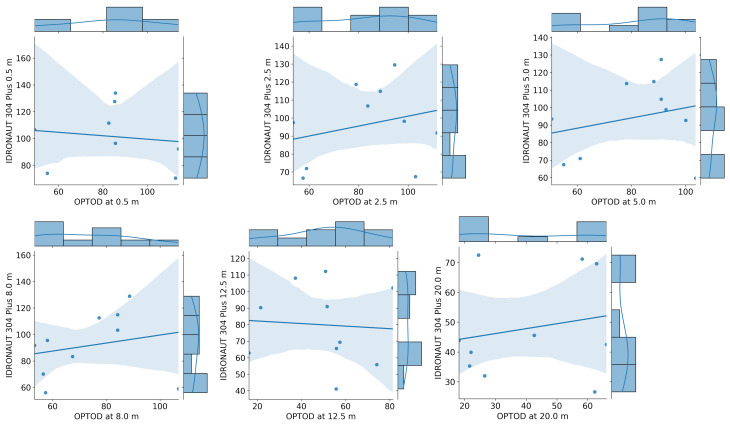
Point to point comparison of dissolved oxygen saturation time series between AHFM and manual sampling with an IDRONAUT 304 Plus probe.

**Figure 20:**
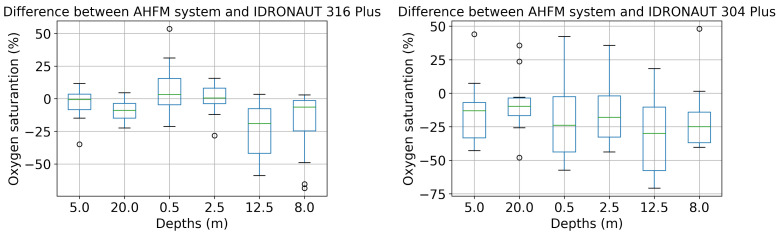
Comparison of FL dissolved oxygen saturation between AHFM time series and manual sampling with IDRONAUT probes.

A point-to-point comparison between the data of the OPTOD chain of sensors and IDRONAUT ’s probes is shown in Figure 18 and Figure 19. The scatter plots are visualized together with the distribution of values. Ideally, if the observations coincided, the points would be arranged on a diagonal. Sensors from 0.4 to 5 m depths have a good correlation with IDRONAUT 316 plus. The dispersion tends to increase when higher values of saturation are registered. There is not a systemic overestimation or underestimation of values, which is therefore evident analysing the scatter plot at 8 m and 20 m depths. In those cases, OPTOD sensors generally underestimate values. The dispersion is higher at 8 m than at 20 m. The data distribution graphs show that values are ranged from about 40% to 160% from 0.4 to 8 m depths, while, at 12.5, from 60% to 100%, and, at 20 m, are even more concentrated between 30% and 70%. While the correlation with IDRONAUT 316 Plus is quite high, the comparison with IDRONAUT 304 Plus does not show same evidence. In fact, a worse correlation must be highlighted. In Figure 19, a high data dispersion and very low correlation is shown at every depth. Such data are also confirmed looking at the boxplot in Figure 20, where variance is very high, median values span from −10% to more than −40% and the interquartile range is also generally quite high in particular at 12.5 m depth. A better performance can be mentioned for the values collected at 20 m. On the contrary, the differences comparison between the AHFM system data and the IDRONAUT 316 Plus shows median values very close to zero at 0.5, 2.5 and 5 m depths. The worse performances are registered at 8 and 12.5 m depths since the interquartile range is respectively about 20% and more than 30%.

**Figure 21:**
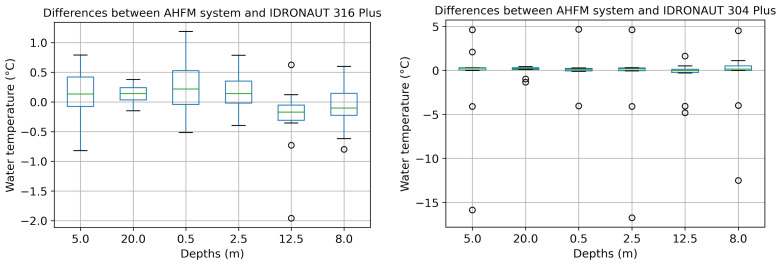
Comparison of water temperatures between AHFM and manual sampling with IDRONAUT probes.

A final consideration concerns the correlation about the water temperature values collected by the analysed systems. To this end, we chose to show just the boxplot representation (Figure 21), which, on one hand, confirms a good interrelation with IDRONAUT 316 Plus; on the other, it shows a much better correlation also with IDRONAUT 304 Plus. In the first case, water temperature differences span between ±1 Celsius degree, with median very close to zero at all depths. In the second, even if the interquartile range is very small and the median is much closer to zero than the previous, more outliers are detected.

## 4. Discussion and Conclusions

In recent years, the monitoring of water bodies is gaining renewed interest because, despite several mitigation actions (e.g., improving the wastewater treatment, banning phosphates from detergents, etc.), they are still susceptible to eutrophication due to climate change effects [49]. In fact, during the last few decades, many climatic parameters are deviating more and more from the average according to the mean values registered since consistent weather observations are available. The World Meteorological Organization (WMO) stated that the most recent years, 2015 to 2021, were likely the seven warmest years ever recorded [50]. In addition, for example, drought events will likely occur more frequently or with a higher intensity either in Europe or in Asia [51,52]. Hence, the protection of water bodies, such as lakes, is crucial since the demand from various categories including drinking water, industrial use, artificial snow production, agriculture, ecological flow requirements, and hydro-power production is increasing more and more [53]. To this end, in Europe and Switzerland, lake protection and water quality control are regulated respectively by the EU Water Framework Directive and by the CIPAIS. Only a low-frequency monitoring that in many cases is not enough to study short-term changes (e.g., algal blooms) is likely more frequent due to previously mentioned factors related to climate change [54] being required. In front of such needs, the digital revolution is an opportunity that can foster the study of new environmental dynamics thanks to the integration of different data sources, the adoption of new technologies for a smarter and high frequent data sampling as well as the breakdown of the digital barriers that still exist in many administrative sectors dedicated to the environmental risk management. In this context, the open digital ecosystem proposed in this article aims at verifying if a solution composed by a new data collection system vendor-agnostic connected to a data management architecture where different data sources are integrated can potentially foster the digitalization of the water sector and help with studying new water dynamics that facilitate decision makers. The data collection implemented concerns an AHFM system based on open technologies (software, hardware) to sample the dissolved oxygen and temperature at different depths. Such system was tested in a real case scenario on Lake Lugano and compared with the samples gained from a traditional campaign which are regularly performed by the local limnologists. Such system demonstrates good reliability and very promising results after one entire year of testing, from 1 January 2021 till 31 December 2021, in harsh environmental conditions, since it was positioned in the middle of the southern part of Lake Lugano, a deep sub-alpine lake. The results of the data completeness showed a very reliable system with a percent of 97.3% with a daily data completeness above 99%. Therefore, the quality assurance performed on the time series collected highlighted how the system did not have critical biases in values or missing data. Almost 97% of the data demonstrated a very high daily quality after testing each value with an increasing level of restriction. However, in the future, more analysis is requested to better investigate the impact of the maintenance activity on the values. In fact, especially during the most productive period of the year (spring and summer), sensors were usually covered by an algae film which could somehow affect the dissolved oxygen measures. Due to the positive results reached, at the time of writing this article, the system was expanded with additional sensors in order to monitor the Chlorophyll-a and Phycocyian concentrations with UNILUX sensors by Chelsea Technologies, in order to have a wider overview on the eutrophication process of the lake and weather parameters with a compact Lufft station. Moreover, in the future, the data collected by this system will be further studied to implement possible new strategies to monitor the lake productivity, which at this moment is based on an effective but expensive and limited methodology, and algal blooms. The AHFM system is mutually connected to the server side open software suite composed of services with different scopes (see Section 2.4) that integrate different data sources, enabling the fully exploitation of the information thanks to the use of the SOS from OGC international standards. In this way, data integration will open up new opportunities for data validation and correlation, as well as new elaborations which can be done by cross analysing the observations. To this end, thanks to this system, it was possible to easily compare and validate the AHFM system with the data of the dissolved oxygen gathered with two types of probes (IDRONAUT 304 Plus, IDRONAUT 316 Plus) that are commonly used during the traditional campaigns performed on the lake. The comparison shows a very good correlation especially with the IDRONAUT 316 Plus showing, on one hand, that the system developed is collecting good quality of data, and on the other hand confirming that the other probe IDRONAUT 304 Plus had some issues probably caused by the membrane disk which needed to be replaced. The open source nature of the software suite developed permitted the adoption of such system by the CNR of Pallanza, a partner of the SIMILE project, which allowed for further individuating bugs and developing new features. This is an important achievement that demonstrates how the openness philosophy can potentially decrease costs, share skills and increase collaborations. In conclusion, the open, cost-effective and replicable digital ecosystem, which was tested and validated for one year in a real case scenario, demonstrates how new technologies can potentially offer an open solution that can on one hand solve the interoperability and help in overcoming the limiting factors (see Section 2.4) that still permeate the monitoring in the water sector.

## Figures and Tables

**Table 1:** Morphometric information about Lake Lugano.

	Total	North Basin	South Basin
Area of the basin (km^2^)	565.6	269.7	295.9
Area (km^2^)	48.9	27.5	21.4
Volume (km^3^)	6.5	4.7	1.8
Mean depth (m)	134	171	85
Max depth (m)	288	288	89

**Table 2:** Quality Indexes list.

QI	Description
0	Raw value erroneous
100	Raw value
101	The value is consistent with the sensors’s range of measurement
102	The value is consistent with the previous one
103	The value is timely consistent
200	The value has been aggregated with less than 60% of valid values
201	The value has been correctly aggregated

**Table 3:** Range test limits used at a data sensing layer.

Observed Property	Range
Water temperature	−10<x<60 (°C)
Oxygen saturation	0<x≤200 (%)
Oxygen concentration	0<x≤20 (mg/L or ppm)

## Data Availability

The data presented in this study are available on request from the corresponding author.
